# Assessment of screening tools for diabetic sarcopenia in type 2 diabetes mellitus: evidence from a scoping review

**DOI:** 10.3389/fendo.2025.1702479

**Published:** 2026-01-20

**Authors:** Jiawei Yin, Xiaotu Zhang, Jing Cai, Hongshi Zhang, Xuefeng Sun, Zilin Wang, Ye Zhang, Lin Li

**Affiliations:** 1 School of Nursing, Changchun University of Chinese Medicine, Changchun, China; 2 Affiliated Hospital to Changchun University of Chinese Medicine, Changchun, China

**Keywords:** diabetic sarcopenia, diagnostic accuracy, endocrinology, scoping review, screening tools

## Abstract

**Objective:**

This study aimed to map and synthesize the available evidence on screening tools for diabetic sarcopenia in patients with type 2 diabetes mellitus (T2DM), highlighting their characteristics, application contexts, and research gaps.

**Methods:**

A comprehensive search was conducted in PubMed, Web of Science, CNKI, and Wanfang Data to identify studies published from 2010 to Deccember 2025. Studies involving adults with T2DM that evaluated screening tools for sarcopenia against established diagnostic criteria (EWGSOP, AWGS, FNIH, or IWGS) were eligible. Two reviewers independently screened studies, extracted data, and assessed methodological quality using the QUADAS-2 tool. Findings were charted and synthesized narratively, with screening tools grouped into functional assessments, anthropometric measures, biomarker-based methods, imaging approaches, and predictive models.

**Results:**

A total of 24 studies with 9,469 participants were included. The most common screening tools were functional assessments, anthropometric measures, biomarkers, and muscle ultrasound. SARC-F showed moderate sensitivity (13.33%-62.63%) and high specificity (67.30%-91.67%), while SARC-CalF improved diagnostic performance. Muscle ultrasound demonstrated high accuracy, with sensitivity ranging from 71.05% to 95.00%. Predictive models with multiple variables (Age, BMI, HbA1c) showed AUC values between 0.800 and 0.932. Challenges included inconsistent cut-off values and limited validation across diverse populations.

**Conclusion:**

Various screening approaches for diabetic sarcopenia have been explored, but no single tool is universally validated for T2DM. Combining functional questionnaires with objective assessments like ultrasound or biomarkers may offer a more practical solution. Future research should focus on standardizing thresholds and testing tools in diverse populations.

## 1 Introduction

Diabetes has emerged as a major global public health challenge, with an estimated 537 million adults affected worldwide as of 2021 (1). In individuals with type 2 diabetes mellitus (T2DM), insulin resistance, chronic inflammation, oxidative stress, and the accumulation of advanced glycation end-products contribute to the progressive decline in muscle mass and function, making patients more susceptible to sarcopenia compared to the general population (2–4).

Studies have shown that the prevalence of sarcopenia in T2DM approaches 20% (5). The duration of diabetes further exacerbates the risk, as longer exposure to hyperglycemia accelerates muscle deterioration, especially in patients with sustained high HbA1c levels (2). Chronic low-grade inflammation, common in T2DM, also contributes to muscle catabolism, with elevated markers such as IL-6 and CRP being closely associated with sarcopenia. Additionally, visceral fat accumulation and obesity, particularly sarcopenic obesity, are significant predictors of muscle loss, as excess fat interferes with muscle maintenance (6). Diabetic complications, including nephropathy and neuropathy, increase the likelihood of sarcopenia by exacerbating both metabolic disruptions and functional impairments (7). Physical inactivity and inadequate dietary intake, particularly insufficient protein and omega-3 fatty acids, compound the risk of sarcopenia in T2DM patients also (5).

Diabetic sarcopenia is strongly associated with an increased risk of falls, functional decline, frailty, and mortality, further leading to a reduction in quality of life and an escalation in healthcare costs (8, 9). Currently, the diagnosis of sarcopenia relies on reference methods such as dual-energy X-ray absorptiometry (DXA) and bioelectrical impedance analysis (BIA) (10), both of which have certain limitations, including cost and limited clinical recognition.

In recent years, various screening tools have been developed to enhance the identification of sarcopenia and reduce reliance on costly imaging techniques (11, 12). However, different screening methods still have their own limitations, necessitating further refinement and validation.

The SARC-F questionnaire is widely used in community screening due to its simplicity (13); however, its sensitivity is relatively low, which has led to the development of SARC-CalF to improve accuracy (14). The Ishii screening test, which combines age, grip strength, and calf circumference, has demonstrated good predictive capability in Asian populations (15). However, it requires additional measurement tools and more time, limiting its application in large-scale population screening (16). Calf circumference measurement, while simple to perform and demonstrating good sensitivity and specificity, may be affected by factors such as edema or other conditions, which could compromise its accuracy (17). The comprehensive screening approach proposed by the European Working Group on Sarcopenia in Older People (EWGSOP2) integrates SARC-F, muscle mass assessment (DXA/BIA), muscle strength testing, and physical function measurement, thereby enhancing sarcopenia detection precision (18). Despite the increasing prevalence of the condition, there is still a lack of specific sarcopenia screening tools for patients with T2DM.

Despite differences in diagnostic criteria across various research groups, significant efforts have been made to standardize and ensure consistency in measurement methods for sarcopenia by international organizations like AWGS, EWGSOP, and SDOC. Voulgaridou (19) emphasize the consistency among these groups regarding key measurement tools such as handgrip strength, gait speed, and muscle mass assessment. Notably, AWGS2019 and EWGSOP 2 have clearly defined handgrip strength and gait speed as core diagnostic criteria for sarcopenia, setting the cutoff values for handgrip strength at <28 kg for men and <18 kg for women, and gait speed at <0.8 m/s as an indicator of low physical performance. The harmonization of these measurement standards provides a foundation for cross-cultural and multi-center studies, ensuring the comparability of diagnostic outcomes.

Furthermore, Bhasin (20) note that SDOC has reached a consensus on the methods for measuring muscle strength and muscle mass, promoting the development of international standards to ensure consistency in global diagnosis. While different groups use tools like DXA and BIA for muscle mass measurement, they have agreed on the definitions and standardization of these tools. SDOC’s efforts ensure that researchers worldwide can apply unified standards and measurement methods, improving the reliability and global applicability of sarcopenia diagnosis. Rapid and accurate selection of screening tools enables early diagnosis and treatment of diabetic sarcopenia, allowing clinicians to promptly develop nutritional and exercise intervention plans, ultimately improving patients’ quality of life (21). Previous reviews have addressed general sarcopenia, but our scoping review uniquely emphasizes the specific screening tools for diabetic sarcopenia in T2DM patients, identifying key gaps in diagnostic accuracy and proposing multimodal screening strategies.

We conducted a scoping review of studies published in the past 15 years to identify gaps in the evidence base that warrant further validation and methodological refinement.

## 2 Materials and methods

### 2.1 Literature searching

**Figure 1:**
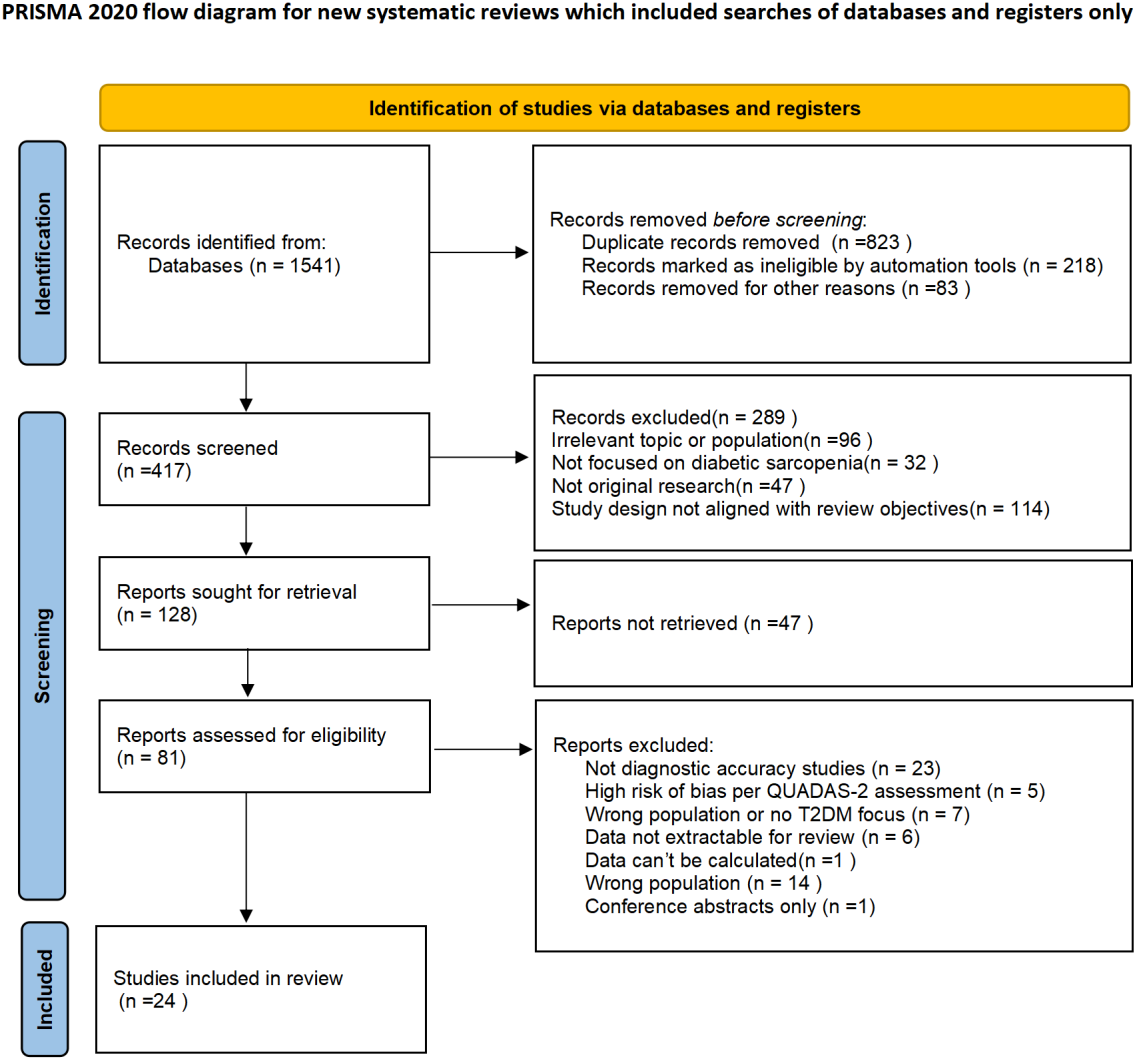
PRISMA flowchart.

To ensure a comprehensive literature search, we systematically searched PubMed, Web of Science, CNKI, and Wanfang databases for relevant studies published between January 2010 and December 2025. Two authors independently conducted the literature search. In cases of disagreement, a third reviewer was consulted to reach a consensus. The detailed study selection process is shown in **Figure 1**.

### 2.2 Literature screening

To minimize selection bias, two trained reviewers independently screened the literature. In case of disagreement, a third reviewer was consulted to reach a consensus.

#### 2.2.1 Inclusion criteria

The PICOS strategy was utilized for the inclusion criteria.

Patient: Adults (≥18 years old) diagnosed with T2DM.
Intervention: Studies that conducted sarcopenia screening.
Comparison: The diagnostic criteria for sarcopenia were derived from the guidelines of EWGSOP, AWGS, FNIH, or IWGS.
Outcome: Studies reporting the accuracy of sarcopenia screening tools, including true positives (TP), false positives (FP), false negatives (FN), and true negatives (TN).
Study Design: Diagnostic test studies.

#### 2.2.2 Exclusion criteria

The following exclusion criteria were applied:

1. Conference abstracts, letters, commentaries, and review articles.
2. Studies with insufficient data where the original authors could not be contacted.
3. Studies involving subjects with major comorbidities such as severe diabetes complications, dialysis, cancer, stroke, psychiatric disorders, or fractures.
4. Studies published in languages other than English or Chinese.

### 2.3 Data extraction

The following data were independently extracted by two authors: author, year, population, sample size, cutoff values of the screening tool, diagnostic criteria for sarcopenia, prevalence, TP, FP, FN and TN. If the information was insufficient, the original authors were contacted via email for clarification.

### 2.4 Literature quality evaluation

We assessed the risk of bias using the QUADAS-2 tool (22), which evaluates four key domains: patient selection, index test, reference standard, and flow and timing. Based on responses to the relevant questions within each domain, the risk of bias was categorized as “low,” “high,” or “unclear.” Two authors independently assessed the quality of the included studies, and the results were presented graphically.

## 3 Results

### 3.1 Characteristics and methodological quality of included studies

#### 3.1.1 Literature screening process

A total of 1541 records were retrieved from four databases. Prior to formal screening, 823 duplicate records were removed. After excluding records that did not meet the eligibility criteria, 417 records remained for further assessment. Ultimately, 24 studies involving a total of 9469 participants met the inclusion criteria and were included in the review.

#### 3.1.2 Characteristics of included studies

We constructed a data extraction table based on the characteristics of the included studies (**Table 1**). Of the 24 studies, 87% were conducted in China, and the average prevalence of sarcopenia across all studies was 28.86%. Three studies used the EWGSOP2 criteria as the diagnostic standard. Further details are provided in **Table 1**.

**Table 1:** Characteristics of the included studies.

Author	Year	Sample size	Screening tool	Cut off	Diagnostic criteria	Prevalence%	TP	FP	FN	TN
Jiang (23)	2024	225	CC*	M34;F33(cm)	AWGS 2019	17.78%	31	16	9	169
							33	42	7	143
Li (24)	2024	225	ucOC*	3.54 ng/ml	AWGS 2019	33.3%	68	75	7	75
Lv (25)	2024	276	CCR*	0.81	AWGS 2019	14.13%	25	55	14	182
			CC	0.437			24	42	15	195
Miao (26)	2024	60	Hcy*+25(OH)D3+IL-6 +TNF-α	–	EWGSOP 2	50%	–	–	–	–
Tang (27)	2023	150	SARC-F	–	AWGS 2019	22.7%	10	12	24	104
			SARC-CalF	–			16	15	18	101
			Finger Ring Test	–			29	24	5	92
Zhang J (28)	2022	160	25(OH)D	13.32 ng/ml	AWGS 2019	50%	50	20	30	60
			PA*	49.2 min/d			64	27	16	53
Zhang Y (29)	2024	105	CC	34.25cm	AWGS 2019	38%	33	23	7	42
			Hip BMD*	0.83cm3			33	26	7	39
He (30)	2023	1125	Prediction Model	12	AWGS 2014	12%	39	204	16	866
Lu (31)	2023	223	Prediction Model	1.77	AWGS 2019	36.3%	63	22	12	126
Simo-Servat (32)	2023	223	MUS*	1.58cm	EWGSOP 2	34%	54	72	21	76
Wei (33)	2023	153	MUS	–	AWGS 2019	24.2%	30	53	7	63
Xu (34)	2022	689	SARC-F	4	AWGS 2019	16.7%	72	191	43	383
			SARC-CalF	11			101	224	14	350
Yu (35)	2023	1131	Prediction Model	–	AWGS 2019	30.06%	266	106	74	685
Akgul (36)	2024	462	SARC-F	4	EWGSOP 2	61.9%	134	55	152	121
			Ishii score	M105;F>120			240	58	46	118
Chen (37)	2022	84	Prediction Model	0.410	AWGS 2019	35.71%	25	9	5	45
Wang (38)	2024	108	MUS	11.4mm	AWGS 2019	55.56%	57	8	3	40
Zhang (39)	2025	523	BMD	–	AWGS 2019	28.02%	71	30	28	237
							30	23	14	90
Wang (40)	2024	1434	Prediction Model	–	AWGS 2019	39.8%	330	125	83	468
							135	51	38	204
Tang (41)	2024	297	Lipoprotein profile*	0.48g/L (FFA*)	AWGS 2019	30%	63	83	26	125
Liu (42)	2025	330	SARC-F	4	AWGS 2019	7.58% to 27.27%	12	20	78	220
			MSRA-7	30			65	173	25	67
			MSRA-5	45			37	53	135	105
			CC	M34;F33			70	123	20	117
			Finger-ring Test	–			53	129	37	111
			SARC-CalF	11			39	60	51	180
			Ishii Score	M105;F>120			72	83	18	157
Zou (43)	2024	1000	Prediction Model	–	AWGS 2019	28.9%	203	152	86	559
Su (44)	2025	157	SARC-F	4	AWGS 2019	44.6%	36	19	34	68
			SARC-CalF	11			64	5	6	82
Laohajaroensombat (45)	2025	329	CC	M34;F33(cm)	AWGS 2019	23.7%	70	123	20	116
			NC*	M38;F32.8(cm)			56	60	34	179
			SARC-F	4			46	52	44	187
			SARC-CalF	11			83	14	7	225
			SARC-F+EBM	12			70	43	20	196
			Chair stand time	12s			72	37	18	202
			HGS*	M28;F18(kg)			65	75	25	164
			Gait speed	1m/s			63	71	27	168
Zhang (46)	2025	225	SARC-F	4	AWGS 2019	24%	10	12	44	159
			SARC-CalF	11			21	16	33	155
			SARC-F+EBM	12			18	13	36	158
			MSRA-5	45			39	76	15	95
			MSRA-7	30			50	117	4	54
			Ishii Score	M105;F>120			45	30	9	141

CC, Calf Circumference; ucOC, undercarboxylated osteocalcin; Hcy, homocysteine; PA, physical activity; MUS, muscle ultrasounds; a-SMI, alternative skeletal muscle index; CCR, serum creatinine/cystatin C ratio; BMD, bone mineral density; NC, Neck Circumference; HGS, Hand Grip Strenth; Lipoprotein profile, The calculation of the lipoprotein profile’s performance metrics (TP, FP, FN, TN) is exemplified by free fatty acids (FFA).

#### 3.1.3 Literature quality evaluation

In this study, the quality of the included diagnostic test studies was systematically assessed using the QUADAS-2 evaluation framework. The results indicated that Akgul (36) demonstrated a low risk of bias across all assessment domains, while the remaining studies exhibited some degree of uncertainty in certain domains. Notably, no studies were identified as high risk.

**Figure 2:**
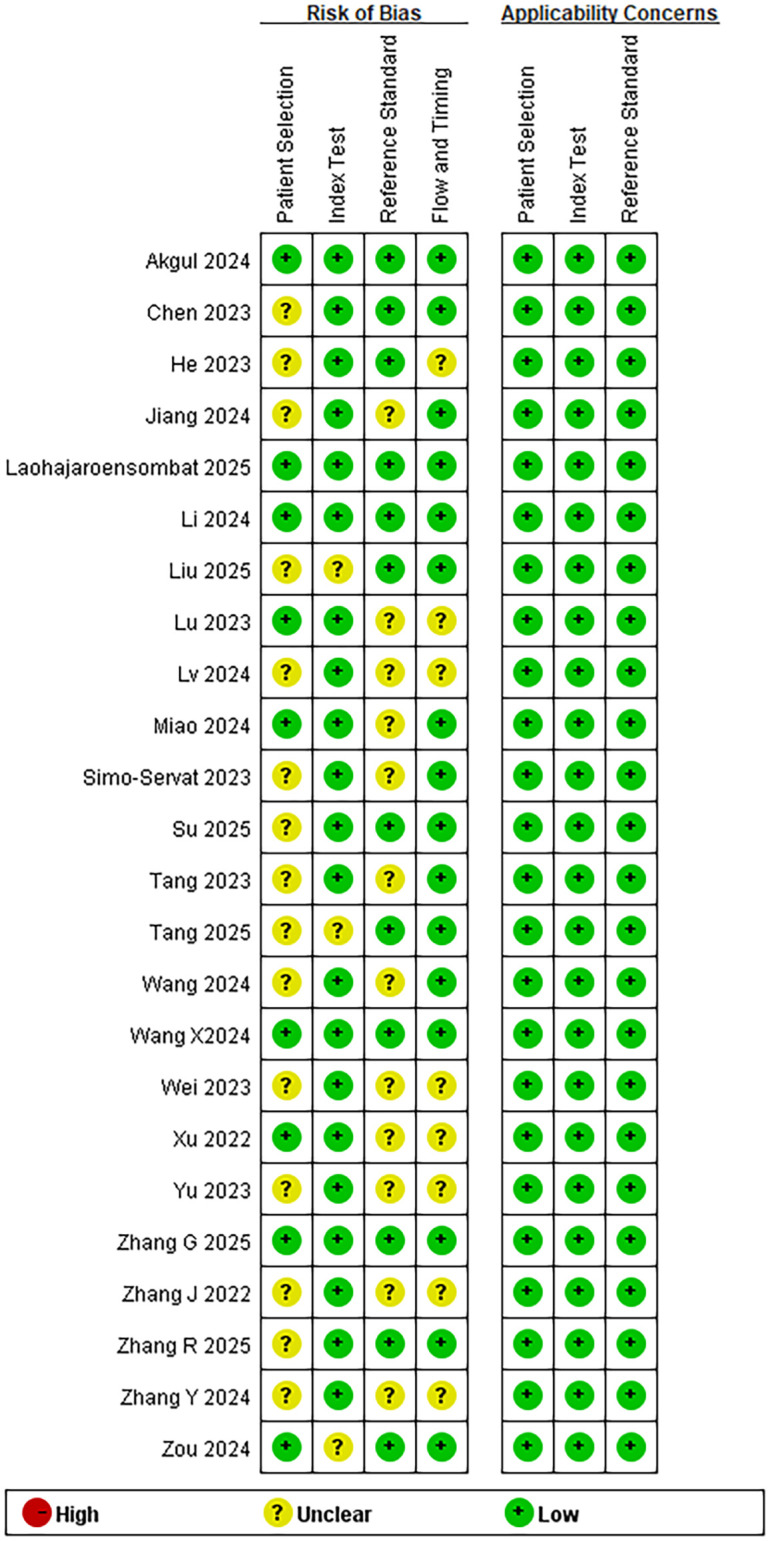
Risk of bias summary.

The risk of bias analysis results for the included studies are presented in **Figure 2**, As it was not clearly specified whether participants were enrolled consecutively or randomly, only five studies (24, 36, 39, 40, 45) in this review was assessed as having a “Low Risk” of bias in the Patient Selection domain. Due to insufficient clarity in the description of follow-up duration and data collection procedures, the consistency of study flow and the appropriateness of data collection timing could not be adequately assessed in eight studies (25, 28–31, 33–35).

### 3.2 Screening tools for diabetic sarcopenia

The diagnostic criteria utilized in each included study, along with the details of corresponding tests, are summarized in **Table 2**.

**Table 2:** Expert consensus on sarcopenia diagnosis: AWGS 2014, AWGS 2019, and EWGSOP2.

Assessment dimensions	Measurement	Description	AWGS2014	AWGS2019	EWGSOP2	
			Cut-off			
Muscle Strength	Handgrip Strength(HGS)	A key measure of upper limb muscle strength, strongly correlated with overall muscle strength, and widely used for screening in elderly and high-risk populations.	Male:<26kg Female:<18kg	Male:<28kg Female:<18kg	Male:<27kg Female:<16kg	
	Chair Stand Test	The chair stand test assesses lower limb strength and endurance, particularly suitable for individuals with limited mobility.	*NA*	>12 s for five rises	>15 s for five rises	
Muscle Mass	DXA	The primary method for measuring muscle mass, offering accurate assessments, but its high cost and equipment requirements limit its use to resource-rich settings.	Male:<7.0kg/m²	Male:<7.0kg/m²	ASM	Male:<20 kg
			Female:<5.4kg/m²	Female:<5.4kg/m²		Female:<15 kg
	BIA	A convenient alternative for large-scale screening, though its accuracy is influenced by factors such as hydration and body fat.	Male:<7.0kg/m²	Male:<7.0kg/m²	ASM /height^2^	Male:<7.0kg/m²
			Female:<5.7kg/m²	Female:<5.7kg/m²		Female:<5.5kg/m²
Physical Function	SPPB	A composite test including gait speed, balance, and chair stand assessments. With a maximum score of 12, a score < 8 indicates poor physical function.	*NA*	≤9 point score	≤8 point score	
	400-meter walk test	Participants were asked to complete 20 laps of 20 meters each, as quickly as possible, with up to two rest breaks allowed during the test.	≤0.8 m/s	≤1 m/s	≤0.8 m/s	
	Timed Up and Go (TUG)test	The TUG test assesses physical performance. Participants are required to stand up from a standard chair, walk to a marker 3 meters away, turn around, walk back, and sit down again.	*NA*	*NA*	≥20s	

### 3.3 Results for the accuracy of screening tools

A total of 24 studies with 9469 participants evaluated a variety of screening tools for diabetic sarcopenia in T2DM (**Table 1**). The screening tools could be categorized into five main groups: functional assessments, anthropometric measures, biomarker-based methods, imaging approaches, and predictive models.

#### 3.3.1 Functional assessment tools

Functional assessments were the most frequently evaluated screening approach. SARC-F was examined in seven studies (27, 34, 36, 42, 44–46), with sensitivity ranging from 13.33% to 62.63% and specificity from 67.30% to 91.67%. The modified SARC-CalF, which incorporates calf circumference measurement, demonstrated improved diagnostic performance compared to SARC-F alone. Across six studies, SARC-CalF showed sensitivity ranging from 38.89% to 91.43% and specificity from 51.52% to 94.25%, with AUC values between 0.693 and 0.980 (27, 34, 42, 44–46).

The Ishii score was evaluated in three studies (36, 42, 46) demonstrating relatively consistent performance with sensitivity ranging from 80.00% to 83.84% and specificity from 65.28% to 82.46%. The AUC values ranged from 0.790 to 0.845, suggesting moderate to good discriminative ability.

The finger-ring test, assessed in two studies (27, 42), showed moderate diagnostic accuracy with sensitivity ranging from 58.89% to 85.29% and specificity from 77.78% to 79.42%.

#### 3.3.2 Anthropometric measures

CC was the most extensively studied anthropometric measure, evaluated in seven studies. However, results were highly heterogeneous. Cut-off values varied considerably across studies (ranging from M34/F33cm to 37cm for males and 36cm for females), with corresponding sensitivity ranging from 60.00% to 90.11% and specificity from 67.80% to 91.36%. When combined with other parameters, diagnostic performance generally improved (25, 29, 42, 45).

The study size, test types, and main findings are shown in **Table 3**, and the summary of sarcopenia diagnostic methods and cutoffs is provided in **Table 4**.

**Table 3:** Summary of study size, screening tests, and main findings.

Author	Sample size	Screening tool	Cut off	Main findings
Jiang (23)	225	CC*	M33.6 F32.3	Men: Sensitivity 78.3%, Specificity 91.4%, Kappa 0.664, PPV 87.3%, NPV 85.2%, AUC = 0.900. Women: Sensitivity 82.4%, Specificity 77.2%, Kappa 0.650, PPV 79.6%, NPV 82.5%, AUC = 0.840.
Li (24)	225	ucOC*	3.54 ng/ml	In elderly T2DM patients, ucOC and tOC levels were significantly higher in the sarcopenia group. ucOC negatively correlated with muscle mass and handgrip strength. Logistic regression showed ucOC as an independent risk factor for sarcopenia (OR = 1.576). UC had a higher diagnostic value for sarcopenia (AUC = 0.79).
Lv (25)	276	CCR*	0.81	CCr was positively correlated with BMI, UCR, T12 CSA, L3 SMI, and calf circumference, and negatively correlated with age, CysC, and ACR. Logistic regression analysis identified BMI, CCr, calf circumference, and ACR as risk factors for sarcopenia. ROC analysis showed that CCr combined with calf circumference had the highest diagnostic value for sarcopenia (AUC = 0.817, sensitivity=91.1%, specificity= 56.4%).
		CC	0.437	
Miao (26)	30	Hcy* 25(OH)D3 IL-6 TNF-α	–	Serum Hcy, IL-6, and TNF-α were higher, while 25(OH)D3 was lower in the sarcopenia group. The combined diagnostic value of Hcy, 25(OH)D3, IL-6, and TNF-α showed high accuracy (AUC = 0.889). Hcy, IL-6, and TNF-α were negatively correlated with walking speed and grip strength, while 25(OH)D3 was positively correlated with these measures.
Tang (27)	150	SARC-F	–	SARC-F: Sensitivity 34.4%, Specificity 90.7%, Positive Predictive Value (PPV) 52.4%, AUC = 0.626. SARC-CalF: Sensitivity 46.9%, Specificity 91.7%, PPV 62.5%, AUC = 0.693. Finger Ring Test: Sensitivity 87.5%, Specificity 77.8%, PPV 53.8%, AUC = 0.826.
		SARC-CalF	–	
		Finger Ring Test	–	
Zhang J (28)	160	25(OH)D	13.32 ng/ml	Sarcopenia group had significantly lower levels of 25(OH)D and PA compared to the T2DM group (*P* < 0.05). Logistic regression identified duration of T2DM, HbA1c, 25(OH)D, and PA as independent factors for sarcopenia (*P* < 0.05). ROC curve analysis showed that 25(OH)D (AUC = 0.720) and PA (AUC = 0.771) were effective in diagnosing sarcopenia in elderly T2DM patients. Severe sarcopenia patients had significantly lower 25(OH)D and PA compared to mild sarcopenia patients (*P* < 0.05).
		PA*	13.32 ng/ml	
Zhang Y (29)	105	CC	34.25cm	Calf circumference and hip BMD were significantly lower in the sarcopenia group compared to the control group (*P* < 0.05). Logistic regression identified calf circumference (OR = 0.592) and hip BMD (OR = 0.002) as significant risk factors for sarcopenia in T2DM patients (*P* < 0.05). ROC curve analysis showed that calf circumference (AUC = 0.811), hip BMD (AUC = 0.722), and the combined index (AUC = 0.863) had high diagnostic value for sarcopenia in T2DM, with the combined index outperforming individual measures.
		Hip BMD*	0.83cm3	
He (30)	1125	Predictive Model	12	The sarcopenia risk score included age, gender, BMI, total energy intake, and proportion of calories from protein. The optimal cutoff was 12 points. In the exploratory population, the AUC was 0.806, with sensitivity of 70.9% and specificity of 81.0%. The external validation showed an AUC of 0.836. The model fit was confirmed by the Hosmer-Lemeshow test (*P* = 0.813).
Lu (31)	223	Predictive Model	1.77	Logistic regression identified male gender, hypertension, BMI, and 25-(OH) VitD as independent factors for sarcopenia. The new a-SMI formula (based on gender, BMI, 25-(OH) VitD, and hypertension had high diagnostic accuracy: AUC = 0.837, sensitivity = 84.0%, specificity = 62.7%. The kappa coefficient between the new approach and AWGS 2019 was 0.669 (*P* < 0.001), showing good agreement.
Simo-Servat (32)	223	MUS*	1.58cm	MUS correlated significantly with BIA (R = 0.4, *P* < 0.02). TMT measured with MUS showed significant inverse correlations with Sarcopenia Risk Index (R = -0.6, P < 0.0001). SARC-F score was inversely correlated with fat-free mass (R=-0.5, *P* < 0.002) and hand-grip strength (R=-0.5, *P* < 0.0002). A TMT < 0.98cm predicted 100% of sarcopenia cases (sensitivity = 100%, specificity = 6.06%).
Wei (33)	153	MUS	–	CSA, MT, and SWE values were significantly lower in sarcopenia patients (*P* < 0.05). ASMI was positively correlated with CSA (r=0.56), MT (r=0.39), and FL (r=0.27) (*P* < 0.001). Handgrip strength was positively correlated with CSA (r=0.45), MT (r=0.25), and SWE (r=0.26). A diagnostic model for sarcopenia using CSA, MT, and SWE had sensitivity of 81.1%, specificity of 75.0%, and AUC = 0.800.
Xu (34)	689	SARC-F	4	Sensitivity: SARC-F ranged from 61.4% to 67.4%, while SARC-CalF ranged from 82.6% to 91.8%. Specificity: SARC-F ranged from 63.1% to 67.3%, and SARC-CalF ranged from 51.5% to 61.2%. AUC for SARC-CalF was consistently higher than SARC-F across all diagnostic criteria, with AUC ranging from 0.74 to 0.81 for SARC-CalF and 0.65 to 0.67 for SARC-F.
		SARC CalF	11	
Yu (35)	1131	Predictive Model	–	Age, sex, BMI, WHR, and heart rate were identified as significant predictors of sarcopenia. The nomogram model incorporating these five variables showed high predictive performance, with AUC = 0.907 (95% CI: 0.890-0.925). Internal validation yielded an AUC = 0.908 (training set) and AUC = 0.904 (testing set). External validation resulted in an AUC = 0.932.
Akgul (36)	462	SARC-F	4	Sensitivity for Ishii score was 84%, and for SARC-F was 47%. Specificity for Ishii score was 67%, and for SARC-F was 69%. PPV for the Ishii score was 79%, and NPV was 45%; for SARC-F, PPV was 71% and NPV was 55%. AUC: Ishii score=0.790, SARC-F=0.598.
		Ishii score	71.8	
Chen (37)	84	Predictive Model	0.410	Sarcopenia group had significantly lower muscle CSA and muscle stiffness compared to non-sarcopenia group (*P* < 0.05). CSA and thickness of the rectus femoris were positively correlated with ASMI and handgrip strength (*P* < 0.001). Logistic regression analysis showed that ΔCSA and ΔSWE were independent predictors of sarcopenia (*P* < 0.05). The diagnostic model combining CSA, SWE, and thickness showed an AUC of 0.883, with 83.3% sensitivity, 83.3% specificity, and 83.3% accuracy.
Wang (38)	108	MUS	11.4mm	MT, pinna angle (PA), and FL were significantly lower in the sarcopenia group compared to the non-sarcopenia group (*P* < 0.05). Sarcopenia predictors: MT was the most significant predictor (OR = 4.576, *P* < 0.001). ROC analysis: The diagnostic accuracy for sarcopenia showed AUC for MT = 0.952, AUC for PA = 0.894, and AUC for SWE = 0.838.
Zhang (39)	523	BMD	–	In middle-aged and elderly men with T2DM, age, HbA1c, and HOMA-IR emerged as independent risk factors for sarcopenia, while elevated lumbar L1–L4 and femoral neck BMD T-values were protective. The DXA-based nomogram model showed solid predictive performance with C-indexes/AUCs of 0.773 (training) and 0.750 (validation), aiding early screening.
Wang (40)	1434	Prediction Model	–	This study developed and validated a 9-factor nomogram predicting sarcopenia in older T2DM patients (AUC = 0.800), identifying age, BMI, diabetes duration, HbA1c, vitamin D, nephropathy, neuropathy, nutritional status, and osteoporosis as key predictors for early screening and intervention.
Tang (41)	297	Lipoprotein profile	0.48g/L (FFA*)	A cross-sectional study of 297 older Chinese T2DM patients (30% with sarcopenia) identified age, BMI, ApoA, lipoprotein, and FFA as independent predictors of sarcopenia in T2DM, with FFA demonstrating the strongest predictive value (AUC = 0.721), suggesting lipoprotein profiles-particularly FFA-may serve as novel biomarkers for early sarcopenia detection and potential therapeutic targets.
Liu (42)	330	SARC-F	4	330 older T2DM patients compared seven sarcopenia screening tools against four diagnostic criteria, finding the Ishii Score had highest accuracy (AUC = 0.79, sensitivity 80%, specificity 65%), while SARC-F showed high specificity (91.67%) but poor sensitivity (13.33%), suggesting CC for rapid screening and SARC-F for confirmatory testing.
		MSRA-7	30	
		MSRA-5	45	
		CC	M34;F33	
		Finger-ring Test	–	
		SARC-CalF	11	
		Ishii Score	M105;F>120	
Zou (43)	1000	Prediction Model	–	Using CHARLS data from 783 diabetic participants, a nomogram was developed incorporating 9 predictors to assess sarcopenia risk, achieving excellent discrimination (AUC 0.808 training, 0.811 internal validation, 0.794 external validation), with age and hemoglobin identified as most significant predictors via machine learning analysis.
Su (44)	157	SARC-F	4	Among 157 T2DM patients aged≥60, SARC-F demonstrated good reliability (Cronbach’s α=0.80, ICC = 0.89) but moderate diagnostic accuracy (AUC = 0.65, optimal cutoff 2.5 with sensitivity 51.4%, specificity 78.2%), while SARC-CalF showed superior performance (AUC = 0.98, optimal cutoff 7.5 with sensitivity 92%, specificity 94%), suggesting SARC-CalF is preferable for sarcopenia screening in T2DM patients.
		SARC-CalF	11	
Laohajaroensombat (45)	329	CC	M34;F33(cm)	Among 329 Thai T2DM outpatients (23.7% sarcopenia prevalence), calf circumference demonstrated highest diagnostic accuracy (AUC = 0.892) with optimized cutoffs of <37cm (males) and <36cm (females) achieving high sensitivity (90.1% males, 91.1% females) and acceptable specificity (77.2% males, 67.8% females). Neck circumference emerged as a promising alternative (AUC = 0.741, cutoffs <39.5cm males, <36.5cm females), while SARC-CalF showed limited sensitivity (48.7%) despite high specificity (93.2%).
		NC*	M38;F32.8(cm)	
		SARC-F	4	
		SARC-CalF	11	
		SARC-F+EBM	12	
		Chair stand time	12s	
		HGS*	M28;F18(kg)	
		Gait speed	1m/s	
Zhang (46)	225	SARC-F	4	In 225 older Chinese T2DM patients (24% sarcopenia prevalence), the Ishii test demonstrated highest screening accuracy (AUC = 0.845, sensitivity 83.33%, specificity 82.46%) with sex-specific performance (males: AUC = 0.855; females: AUC = 0.863), while SARC-F showed low sensitivity (18.52%) despite high specificity (92.98%), and SARC-CalF improved performance (AUC = 0.834, sensitivity 38.89%, specificity 90.64%).
		SARC-CalF	11	
		SARC-F+EBM	12	
		MSRA-5	45	
		MSRA-7	30	
		Ishii Score	M105;F>120	

SWE, Shear Wave Elastography; ApoA, apolipoprotein.

**Table 4:** Summary of sarcopenia diagnostic methods and cutoffs.

Test	Measurement	Cutoff	Advantages	Disadvantages
CC* (47)	Measured at the widest point of the calf using a non-elastic tape.	Male:< 34cm Female:< 33cm	Low-cost, easy, correlates with muscle mass and strength, suitable for large-scale screening.	Lower sensitivity and specificity than advanced methods (DXA/MRI), affected by edema or obesity, and lacks universal cutoff.
ucOC (48, 49)	Measured in serum to assess undercarboxylated osteocalcin, a marker of bone turnover.	No universal cutoff; elevated levels indicate muscle loss.	Non-invasive, easy to measure, correlates with muscle mass, bone health, and metabolism.	②Influenced by bone turnover, vitamin K status, and kidney function. ①Limited large-scale human data.
CCR (50, 51)	Calculated from serum creatinine divided by cystatin C from fasting blood.	No universal cutoff; higher values correlate with greater muscle mass.	①Low-cost, non-invasive, easy to implement. ②Correlates with muscle mass and strength. ③Useful when DXA is unavailable.	①Moderate diagnostic accuracy (AUC ~ 0.78). ②Affected by renal function, hydration, and diet. ③No universally accepted cutoff.
SARC-F (14, 52)	A 5-item self-reported questionnaire assessing physical function and strength.	Total score≥4 indicates risk of sarcopenia.	Low-cost, easy and rapid to administer, requires no specialized equipment, suitable for large-scale or resource-limited settings.	①Low to moderate sensitivity. ②Limited diagnostic accuracy when used alone, especially in populations with early-stage sarcopenia.
SARC-CalF	Combines SARC-F with calf circumference measurement.	Similar to SARC-F; combination improves diagnostic accuracy.	①Simple, low-cost, non-invasive. ②Improved diagnostic accuracy compared to SARC−F alone. ③Suitable for large-scale screenings.	①Moderate sensitivity and specificity. ②Performance varies by population and cutoffs. ③Cannot replace direct measures of muscle mass or strength.
Finger Ring Test (53)	The test involves encircling the calf with the thumb and index finger.	Non-overlapping fingers indicate sarcopenia risk.	①Simple, low−cost, non−invasive. ②Easy to administer, requires no equipment.	①Risk of false negatives. ②Cannot quantify muscle mass or strength directly.
25(OH)D (54, 55)	Measured in serum; reflects vitamin D status for bone and muscle health.	Levels < 20 ng/mL linked to increased sarcopenia risk.	Simple, low-cost biomarker for screening; correlates with muscle mass and strength.	Mixed evidence on supplementation benefits; influenced by multiple factors like comorbidities and physical activity.
PA (56)	Assessed by questionnaires or accelerometers for physical activity tracking.	No universal cutoff; 150 minutes of moderate activity weekly is recommended.	Non-invasive, accessible, cost-effective; improves muscle mass and function.	Moderate evidence; effects vary by age and baseline health.
MUS (57)	Uses ultrasound to measure muscle thickness, cross-sectional area, and fascicle length.	No universal cutoff; CSA and MT used for diagnosis.	Non-invasive, portable, low-cost, correlates well with DXA/BIA.	①Lacks standardization and uniform cutoffs.
Hip BMD (58)	Measured by DXA, yielding areal BMD at spine, hip, or femoral neck.	T-score≤-2.5 defines osteoporosis; correlated with sarcopenia.	Gold standard for bone health; objective, quantitative; useful for osteosarcopenia assessment.	Does not assess muscle mass or strength directly; variability in sites; only associative with sarcopenia.
Ishii score (59)	A score calculated from age, hand-grip strength, and calf circumference.	≥105 for males ≥120 for females.	Low-cost; requires only simple clinical measures (grip strength, calf circumference, age); shows good screening performance (AUC often > 0.80).	Cutoffs may vary by population; not a direct measure of muscle mass/strength.

NC was evaluated as an alternative measure in one study (45), demonstrating moderate accuracy with sensitivity of 62.22% and specificity of 74.90% (AUC = 0.741).

#### 3.3.3 Biomarker-based methods

Serum biomarkers demonstrated variable diagnostic performance. Single biomarker approaches included: ucOC (24): sensitivity 90.67%, specificity 50.00%, AUC = 0.790; CCR (25): sensitivity 64.10%, specificity 76.79%, AUC = 0.780; 25(OH)D (28): sensitivity 62.50%, specificity 75.00%, AUC = 0.720. Multi-biomarker panels showed enhanced performance. Miao et al. (26) evaluated a combined panel of homocysteine, 25(OH)D_3_, IL-6, and TNF-α, achieving an AUC of 0.889, though specific sensitivity and specificity values were not reported. Tang (41) examined lipoprotein profiles, particularly FFA, demonstrating sensitivity of 70.79% and specificity of 60.10% (AUC = 0.721).

#### 3.3.4 Imaging approaches

MUS was evaluated in three studies (32, 33, 38). Cut-off values varied across studies (1.58cm, 11.4mm). Sensitivity ranged from 71.05% to 95.00%, specificity ranged from 51.35% to 84.30%, and AUC values ranged from 0.690 to 0.952.

BMD was assessed in two studies. In Zhang Y et al. (29), a cut-off of 0.83cm³ yielded sensitivity of 82.50%, specificity of 60.00%, and AUC of 0.722. Zhang G et al. (39) reported AUC values ranging from 0.750 to 0.773 for BMD-based screening.

#### 3.3.5 Predictive models and nomograms

Six studies developed predictive models incorporating multiple clinical variables. He et al. (30) reported an AUC of 0.806 in the training set and 0.836 in the validation set, with sensitivity of 70.9% and specificity of 81.0%. Lu et al. (31) achieved an AUC of 0.837 with sensitivity of 84.0% and specificity of 62.7%. Yu et al. (35) demonstrated the highest overall performance with AUC values of 0.907 (95% CI: 0.890-0.925) in the initial analysis, 0.908 in the training set, 0.904 in the testing set, and 0.932 in external validation. Chen et al. (37) reported an AUC of 0.883 with sensitivity and specificity both at 83.3%. Wang et al. (40) achieved an AUC of 0.800, while Zou et al. (43) reported AUC values of 0.808 in the training set, 0.811 in internal validation, and 0.794 in external validation. Common predictors incorporated across these models included age, gender, BMI, diabetes duration, HbA1c, vitamin D levels, presence of diabetic complications, nutritional status, and osteoporosis.

#### 3.3.6 Summary of diagnostic performance

**Figure 3:**
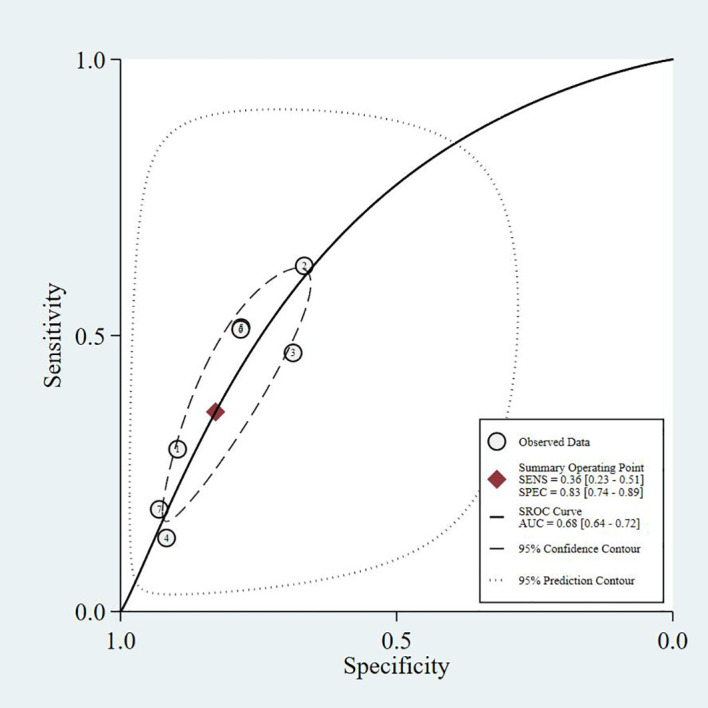
SARC-F - SROC curve.

**Figure 4:**
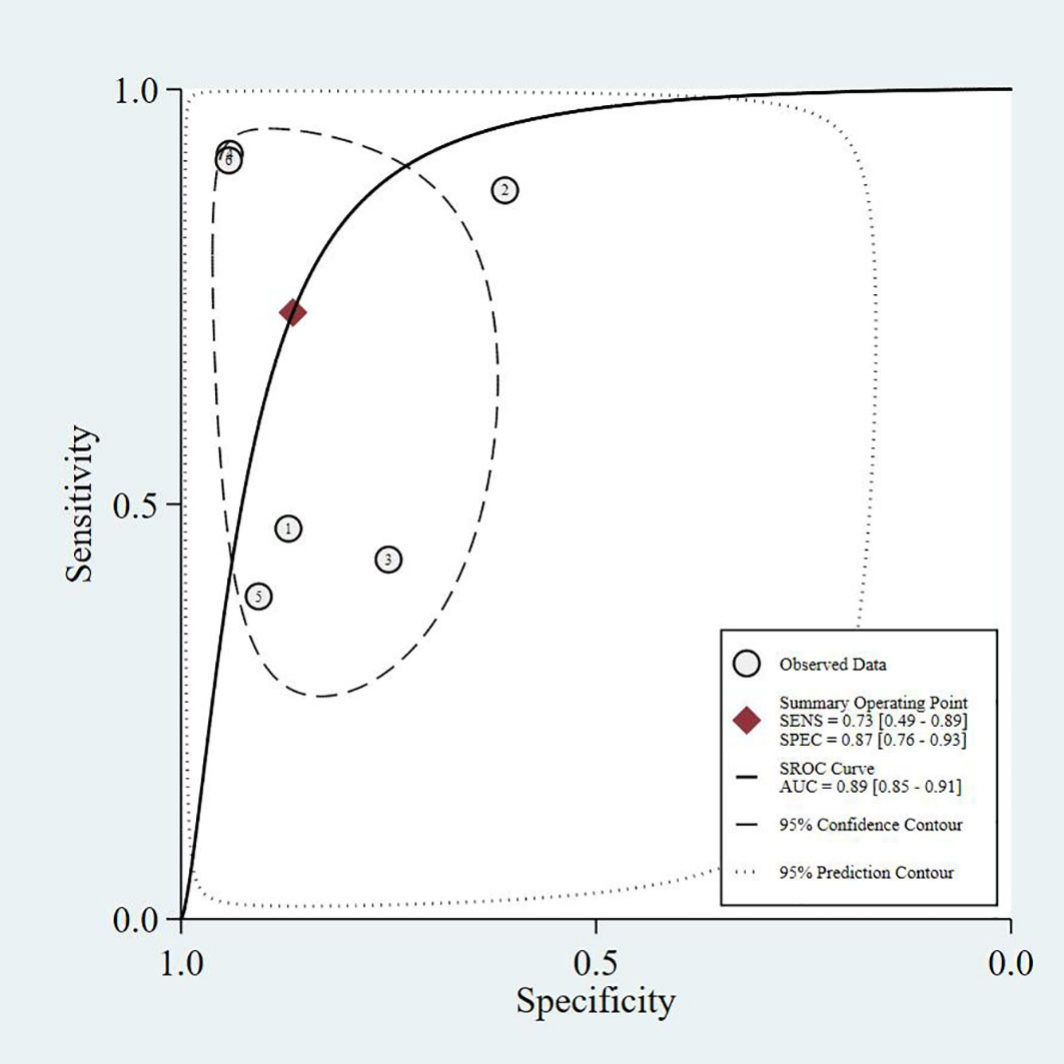
SARC-CalF - SROC curve.

**Figure 5:**
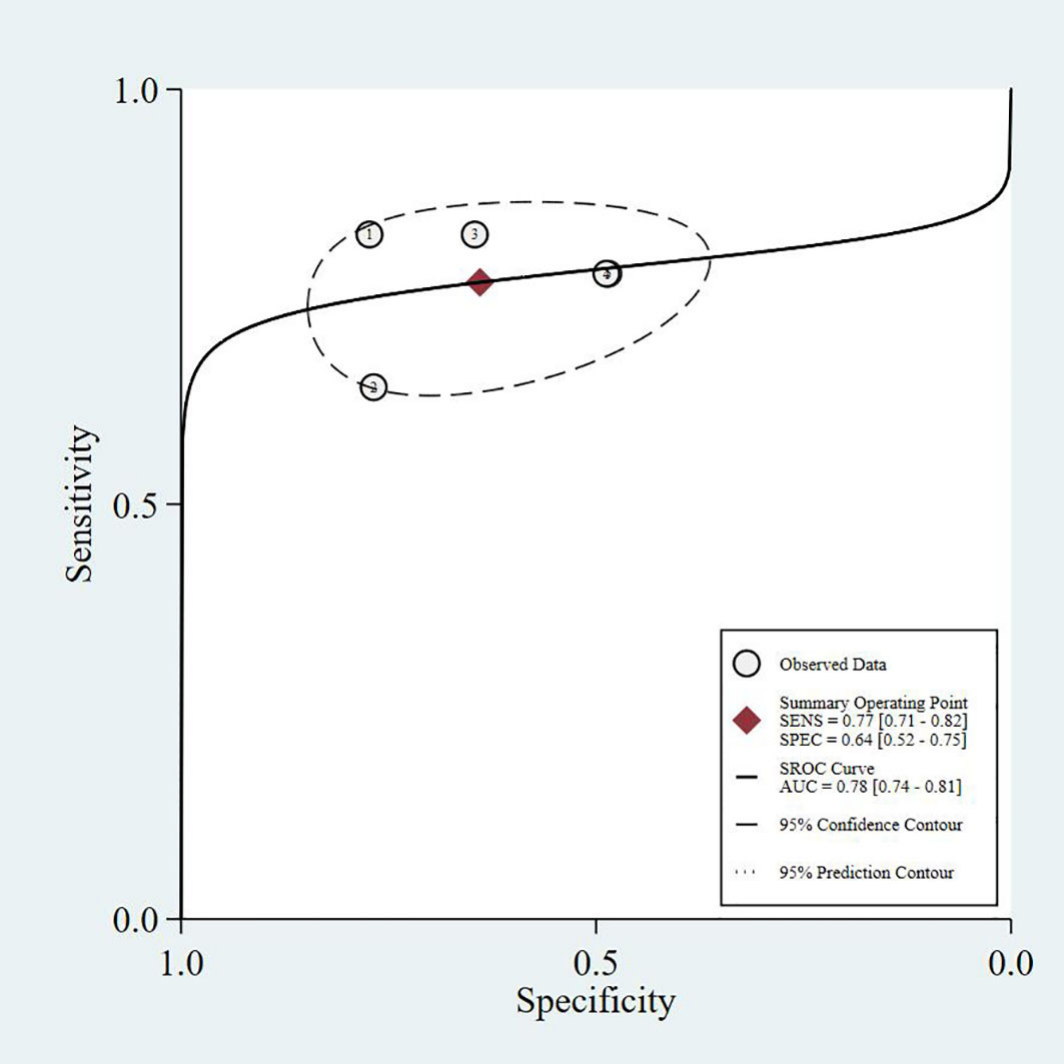
CC - SROC curve.

**Figure 6:**
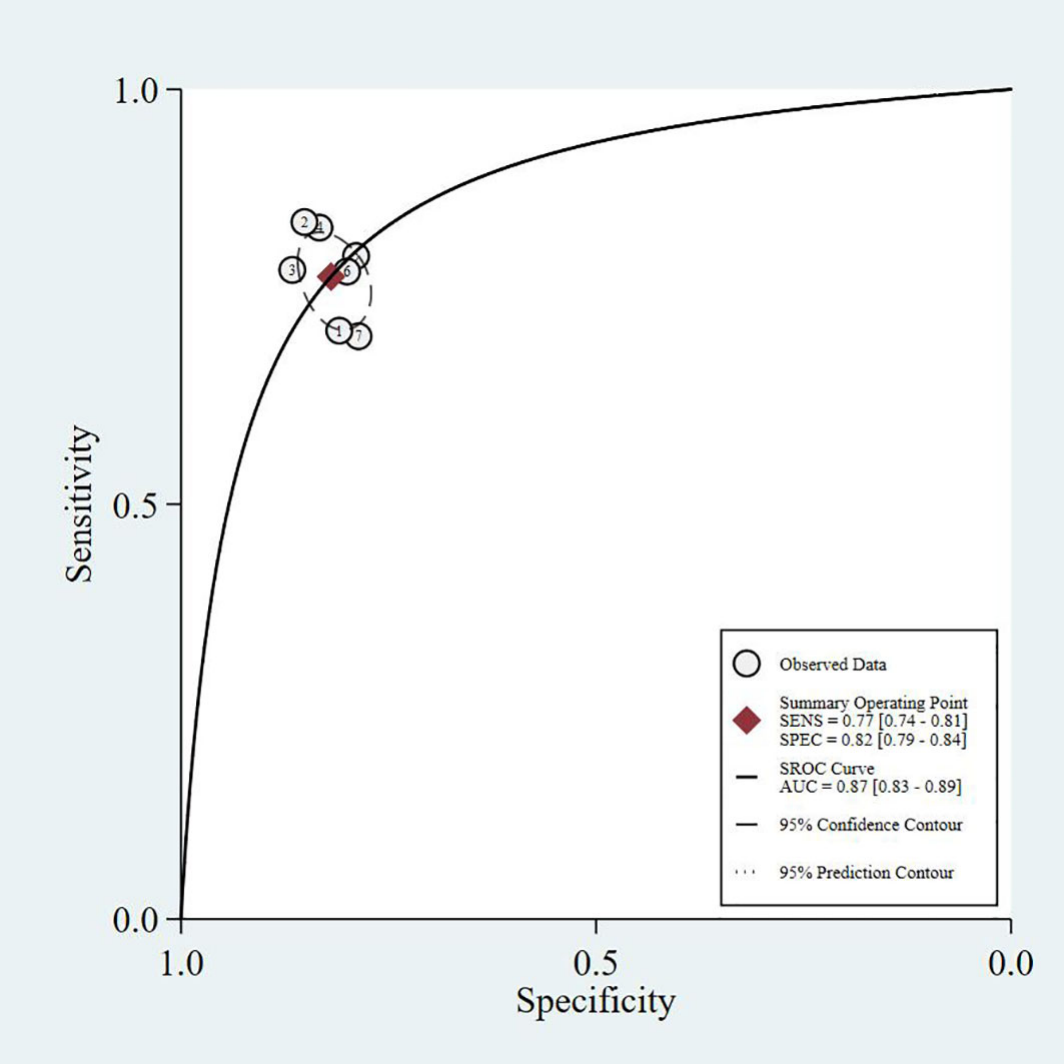
Prediction Model - SROC curve.

This study summarizes the SROC curves of the most commonly reported screening tools, as shown in **Figures 3**–**6**.

The four SROC curves show that multimodal prediction models outperform single indicators in diagnosing diabetic sarcopenia. The SARC-F tool has moderate accuracy (AUC = 0.77), while SARC-CalF improves performance (AUC = 0.80). CC shows lower diagnostic ability (AUC = 0.75). In contrast, the Prediction Model integrating multiple clinical variables demonstrates the highest accuracy (AUC = 0.83), highlighting that combining multiple factors leads to more reliable and stable results.

## 4 Discussion

### 4.1 Current screening methods in diagnosing diabetic sarcopenia

This study demonstrates that current screening methods for diabetic sarcopenia include SARC-F, SARC-CalF, the Ishii screening test, the finger-ring test, and calf circumference measurement. Researchers have employed serum biomarkers such as 25(OH)D or ultrasound-based assessments also. Currently, there are no sarcopenia screening tools specifically designed and validated exclusively for patients with T2DM. In T2DM patients, metabolic abnormalities such as muscle fat degeneration, insulin resistance, and elevated blood glucose levels contribute to muscle catabolism (6), highlighting the need for targeted screening tools.

Muscle fat infiltration represents a key mechanism linking T2DM to sarcopenia, with a positive correlation between intermuscular adipose tissue, intramyocellular lipids, and skeletal muscle insulin resistance (60). Pro-inflammatory cytokines released by adipose tissue and circulating free fatty acids directly disrupt insulin signaling, impairing insulin’s ability to regulate glucose metabolism in skeletal muscle (61). Quantitative magnetic resonance imaging studies have revealed higher intramuscular and intermuscular fat infiltration in the quadriceps of T2DM patients, along with significantly lower isokinetic muscle strength compared to healthy controls (62).

Most screening approaches use the general sarcopenia screening tools (SARC-F, SARC-CalF, Ishii screening test) in diabetic populations, there is growing recognition that T2DM patients may require modified screening approaches due to their unique pathophysiology (63).

### 4.2 Diagnostic performance of screening tools

Based on the 23 included studies with 9,469 participants, we systematically evaluated the diagnostic performance of different screening approaches. The following sections discuss the accuracy, advantages, and limitations of each tool category.

#### 4.2.1 Functional assessment tools

Functional assessments were the most frequently evaluated screening approach in our review, examined in 13 studies. SARC-F, assessed in seven studies (27, 34, 36, 42, 44–46) showed moderate sensitivity (13.33%-62.63%) and high specificity (67.30%-91.67%), with AUC values ranging from 0.598 to 0.77. The modified SARC-CalF, which incorporates calf circumference measurement, demonstrated improved diagnostic performance across six studies, with sensitivity ranging from 38.89% to 91.43%, specificity from 51.52% to 94.25%, and AUC values between 0.693 and 0.980.

The Ishii score, evaluated in three studies (36, 42, 46), demonstrated relatively consistent performance with sensitivity ranging from 80.00% to 83.84%, specificity from 65.28% to 82.46%, and AUC values from 0.790 to 0.845, suggesting moderate to good discriminative ability. The finger-ring test, assessed in two studies (27, 42), showed moderate diagnostic accuracy with sensitivity ranging from 58.89% to 85.29% and specificity from 77.78% to 79.42%.

The superior performance of SARC-CalF compared to SARC-F alone suggests that combining subjective functional assessment with an objective anthropometric measure enhances diagnostic accuracy in T2DM patients. However, the wide ranges in sensitivity observed across studies indicate potential population-specific factors that warrant further investigation.

#### 4.2.2 Anthropometric measures

CC was the most extensively studied anthropometric measure in our review, evaluated in seven studies (23, 25, 27). (29, 42, 45, 46) However, results were highly heterogeneous. Cut-off values varied considerably across studies (ranging from M34/F33cm to M37/F36cm), with corresponding sensitivity ranging from 60.00% to 90.11% and specificity from 67.80% to 91.36%. For instance, Lv (25) found that CC yielded relatively low sensitivity (61.54%) in T2DM patients when used alone, which may be attributable to confounding factors such as edema or varicose veins common in diabetic populations. However, Jiang (23) observed better diagnostic performance (sensitivity 78.3%-82.4%, specificity 77.2%-91.4%) with sex-specific cutoffs. Geographic variation may also contribute to these differences, as the prevalence of T2DM and associated complications like peripheral neuropathy varies across regions. When combined with other parameters, diagnostic performance generally improved (25, 29, 41, 45) suggesting that CC may be more valuable as part of a multimodal screening approach.

NC was evaluated as an alternative measure in one study (45), demonstrating moderate accuracy with sensitivity of 62.22%, specificity of 74.90%, and AUC of 0.741. While NC offers potential as a convenient screening tool, the limited evidence from our review prevents definitive conclusions about its utility in diabetic sarcopenia screening.

#### 4.2.3 Biomarker-based approaches

With the deepening of research on metabolic abnormalities in T2DM, an increasing number of biomarkers have been investigated for their potential role in screening diabetic sarcopenia. Elevated nocturnal cortisol levels were found to be significantly associated with sarcopenia risk and outperformed several traditional clinical indicators in predictive ability (64).

Metabolomic and lipidomic analyses further revealed sarcopenia-specific alterations in circulating metabolites. Hsu et al. identified 12 plasma metabolites with significant differences between sarcopenia and non-sarcopenia groups, including decreased isoleucine and creatinine and increased phosphatidylinositol species, among which PI 32:1 showed the highest discriminative value (65).

Another study reported that 82 metabolites were significantly altered in patients with diabetic sarcopenia, with N,N-dimethylarginine and 5′-methylthioadenosine demonstrating strong predictive potential (66).

Lipid-related indices have also been linked to sarcopenia. Yin et al. showed that lipid ratios, including non-HDL-C/HDL-C, TG/HDL-C, LDL-C/HDL-C, and RC/HDL-C, were significantly correlated with sarcopenia risk, with RC/HDL-C displaying the strongest association (67).

In the present review, several serum biomarkers were evaluated. Miao (26) reported that a combined panel of homocysteine, 25(OH)D_3_, IL-6, and TNF-α achieved high sensitivity but relatively low specificity. Lv (26) demonstrated that the CCR alone had limited diagnostic sensitivity, although performance improved when combined with calf circumference. Li (26) examined serum osteocalcin; however, data instability affected the interpretation of diagnostic performance.

The CCR serves as a serum biomarker for predicting muscle mass in patients with chronic kidney disease (68) and can also be used to calculate the muscle reduction index to predict new-onset diabetes (69). This finding is consistent with the results of another meta-analysis, which demonstrated that CCR has a certain degree of accuracy in predicting sarcopenia. When diagnosing sarcopenia based on five different diagnostic criteria, it exhibited a pooled sensitivity ranging from 51% (95%CI 44-59%) to 86% (95%CI 70-95%) and a pooled specificity ranging from 55% (95%CI 38-70%) to 76% (95%CI 63-86%) (70). In the present study, Lv (25) demonstrated that CCR screening for sarcopenia in T2DM patients exhibited relatively low sensitivity; however, diagnostic accuracy improved significantly when combined with calf circumference.

Previous research has confirmed the relationship between β2-microglobulin (β2-MG) and T2DM cardiovascular disease, diabetic nephropathy (71), and microvascular complications (72). Recent studies have discovered that β2-MG can induce myotube atrophy by inhibiting integrin β1 expression through intracellular reactive oxygen species, resulting in impaired FAK/AKT/ERK signaling pathways while enhancing nuclear translocation of FoxO transcription factors, thereby exerting detrimental effects on muscle metabolism.

T2DM is frequently associated with elevated β2-MG levels; therefore, enhanced detection of serum biomarkers for sarcopenia in T2DM patients is necessary to advance large-scale sarcopenia screening initiatives.

#### 4.2.4 Imaging approaches

SWE, specifically SWEstraight, has been utilized to measure tissue elasticity and assess muscle stiffness in patients with T2DM (73). Wei (33) demonstrated that SWE reliably reflects changes in muscle quality in T2DM patients, with an AUC of 0.762 (95%CI: 0.643-0.882) and a sensitivity of 82.8%. Similar studies have shown that patients with sarcopenia exhibit significantly lower SWE relaxation, SWE tension, and ΔSWE values compared to non-sarcopenic individuals, indicating a reduction in muscle stiffness and elasticity in the former group (38). Furthermore, the study identified MT as the most important predictor of sarcopenia, with an AUC of 0.952, and found that when MT ≤ 11.4 mm, the sensitivity was 95.0% and specificity was 84.3%.

However, the study by Simo-Servat (32) reported lower sensitivity and specificity, which may be attributed to the characteristics of the study cohort, comprising T2DM patients with an average age of 77.72 ± 5.08 years and a higher average BMI (31.19 ± 6.65 kg/m²). This suggests that obesity could interfere with ultrasound measurements of muscle thickness due to the confounding effects of adipose tissue, thereby further affecting the assessment of muscle quality. Additionally, the study found that participants with higher BMI may experience increased errors in BIA, as BIA is susceptible to variations in hydration status and body fat percentage, both of which could also influence the accuracy of muscle ultrasound measurements (74).

Additionally, research investigating the relationship between bedside ultrasound measurements of quadriceps thickness and gait parameters and sarcopenia revealed that quadriceps thickness correlates with walking speed, gait stability, and sarcopenia risk (75), suggesting that ultrasound may serve as an effective screening tool for muscle loss in T2DM patients.

#### 4.2.5 Predictive models and nomograms

Six studies in our review developed predictive models incorporating multiple clinical variables (30, 31, 35, 37, 40, 43) demonstrating generally superior diagnostic performance compared to single screening tools. AUC values ranged from 0.800 to 0.932 across different validation cohorts. He et al. (30) reported an AUC of 0.806 in the training set and 0.836 in the validation set, with sensitivity of 70.9% and specificity of 81.0%. Yu et al. (35) demonstrated the highest overall performance with AUC values of 0.907 (95% CI: 0.890-0.925) in the initial analysis and 0.932 in external validation.

Common predictors incorporated across these models included age, gender, BMI, diabetes duration, HbA1c, vitamin D levels, presence of diabetic complications (particularly nephropathy and neuropathy), nutritional status, and osteoporosis. The consistent inclusion of diabetes-specific variables (HbA1c, diabetes duration, diabetic complications) across multiple models underscores the importance of considering T2DM-related factors in sarcopenia risk assessment. As shown in our SROC curve analysis (**Figures 3**-**6**), multimodal prediction models outperformed single indicators, with the prediction model achieving the highest AUC (0.83) compared to SARC-F (0.77), SARC-CalF (0.80), and CC (0.75).

The superior performance of these predictive models likely reflects their ability to capture the multifactorial nature of diabetic sarcopenia. However, their clinical implementation requires consideration of feasibility, as some models incorporate multiple measurements that may not be readily available in all clinical settings. Additionally, most models were developed and validated in Chinese populations, necessitating external validation in diverse ethnic and geographic cohorts before widespread adoption.

### 4.3 Impact of confounders in sarcopenia diagnosis

Several of the studies included in our analysis reported a significant presence of diabetic nephropathy and diabetic peripheral neuropathy in the patient population. For example, He noted that 38.6% of participants in their exploratory population had diabetic nephropathy, while 42.5% reported having diabetic peripheral neuropathy (30). Similarly, in the study by Wei (33), 14.8% of patients with sarcopenia had diabetic nephropathy, and 56.8% had peripheral neuropathy. This high prevalence of renal and neuropathic complications in T2DM patients complicates the interpretation of sarcopenia diagnoses, as both conditions may obscure the true extent of muscle loss. Except for the three studies mentioned, the other studies included in this report did not report these comorbidities, as they were excluded based on the established exclusion criteria.

Beyond kidney disease and neuropathy, other comorbidities and conditions-including obesity, malnutrition, chronic inflammation, multimorbidity burden, low physical activity and metabolic disorders-also constitute important confounders for sarcopenia diagnosis, as they independently influence muscle mass, strength and function. For instance, recent large-scale data show that individuals with sarcopenia or sarcopenic obesity exhibit significantly higher prevalence of multimorbidity compared with non-sarcopenic peers (76).

These factors may bias assessments of muscle decline by contributing to muscle wasting through inflammation, hormonal imbalance, undernutrition or fat infiltration or by masking sarcopenia under obesity or poor function (77).

### 4.4 Future research directions

Future research should focus on standardizing diagnostic thresholds and developing more accurate multimodal screening tools that combine subjective assessments like SARC-F with objective measures such as ultrasound and biomarkers. Longitudinal studies are needed to assess prognostic value, while external validation in diverse populations is essential for generalizability. Additionally, exploring novel biomarkers and advanced imaging techniques like shear wave elastography may improve early detection and management of diabetic sarcopenia.

### 4.5 Limitations

This review has several limitations. First, the use of varying reference standards for sarcopenia hindered valid pooling of sensitivity and specificity. Second, the cross-sectional nature of most studies limits assessment of longitudinal or prognostic performance. Third, heterogeneous thresholds for index tests and diverse settings complicate comparisons. Finally, The dominance of Chinese cohorts and hospital-based populations may introduce geographic bias, limiting the generalizability of findings to other regions and community settings. Hospital-based studies may overestimate the utility of some screening tools. Additionally, while multimodal screening approaches show promise, their readiness for widespread use remains uncertain without further standardized validation in diverse populations.

## 5 Conclusion

This review summarizes and analyzes the types and accuracy of existing diabetic sarcopenia screening tools. Comparing the diagnostic accuracy of subjective and objective screening tools for T2DM patients reveals that combining traditional sarcopenia screening tools with objective examinations such as serum biomarkers or ultrasound is more suitable for diagnosing diabetic sarcopenia.

## Data Availability

The original contributions presented in the study are included in the article/supplementary material. Further inquiries can be directed to the corresponding author.
